# Supplementary material for: A Critical View of the Application of the APEX Software (Aqueous Photochemistry of Environmentally-Occurring Xenobiotics) to Predict Photoreaction Kinetics in Surface Freshwaters
Source: Molecules. 2019 Dec 18;25(1):9. doi: 10.3390/molecules25010009 (PMC7017383; doi:10.3390/molecules25010009)
Supplement: Supplementary file 1 [file molecules-25-00009-s001.zip › 3.2.4_gcc-4.4.0.zip!/3.2.4_gcc-4.4.0/3.2.4_gcc-4.4.0/doc/octave/html/interpreter/Killing-and-Yanking.html]

Killing and Yanking - Untitled


Next: Commands For Text,
Previous: Cursor Motion,
Up: Command Line Editing


---

#### 2.4.2 Killing and Yanking

Killing text means to delete the text from the line, but to save
it away for later use, usually by yanking it back into the line.
If the description for a command says that it `kills' text, then you can
be sure that you can get the text back in a different (or the same)
place later.

Here is the list of commands for killing text.

`C-k`: Kill the text from the current cursor position to the end of the line. `M-d`: Kill from the cursor to the end of the current word, or if between words, to the end of the next word. `M-<DEL>`: Kill from the cursor to the start of the previous word, or if between words, to the start of the previous word. `C-w`: Kill from the cursor to the previous whitespace. This is different than `M-<DEL>` because the word boundaries differ.

And, here is how to yank the text back into the line. Yanking
means to copy the most-recently-killed text from the kill buffer.

`C-y`: Yank the most recently killed text back into the buffer at the cursor. `M-y`: Rotate the kill-ring, and yank the new top. You can only do this if the prior command is `C-y` or `M-y`.

When you use a kill command, the text is saved in a kill-ring.
Any number of consecutive kills save all of the killed text together, so
that when you yank it back, you get it in one clean sweep. The kill
ring is not line specific; the text that you killed on a previously
typed line is available to be yanked back later, when you are typing
another line.
